# Supplementary material for: Base-resolution prediction of transcription factor binding signals by a deep learning framework
Source: PLoS Comput Biol. 2022 Mar 9;18(3):e1009941. doi: 10.1371/journal.pcbi.1009941 (PMC8982852; doi:10.1371/journal.pcbi.1009941)
Supplement: S1 Text — (DOCX) [file pcbi.1009941.s001.docx]

**S1 Text. A brief description of applying FCNA^*^ to predict motifs on negative sequences**

The framework of FCNA^*^ is symmetrical, which consists of a top-down encoding process, a bottom-up decoding process. In the FCNA method, the top-down encoding process is used to encode the feature maps and the bottom-up decoding process is used to restore the size of feature maps. Besides, skip lines are employed to combine the nucleotide position information in the encoder with the semantic information in the decoder, and the hard negative mining loss is adopted to alleviate the problem of imbalanced data. FCNA^*^ takes DNA sequences as input and uses the base-resolution labels (0/1) which were annotated by position counting matrices (PCMs) collected from the HOCOMOCO database.

We used 10 TF datasets in the A549 cell line from the original paper and employed two ways of generating negative sequences: 1) directly using the upstream sequences of positive sequences; 2) using the shuffled dinucleotides of positive sequences. Then, following the same process as the one for positive sequences, we used FCNA^*^ to predict motifs on the above negative sequences (The details can refer to the original paper). The experimental results are shown in S5 Fig, and we can see that the performance of FCNA^*^ predicting motifs on the negative sequences is almost equal to that on the positive sequences. This observation demonstrates that FCNA^*^ is extremely dependent on the PCMs so that it cannot distinguish positive sequences from negative sequences, which means that it even using the negative sequences can also get very good results.
