# Supplementary figures and images for: Base-resolution prediction of transcription factor binding signals by a deep learning framework
Source: PLoS Comput Biol. 2022 Mar 9;18(3):e1009941. doi: 10.1371/journal.pcbi.1009941 (PMC8982852; doi:10.1371/journal.pcbi.1009941)

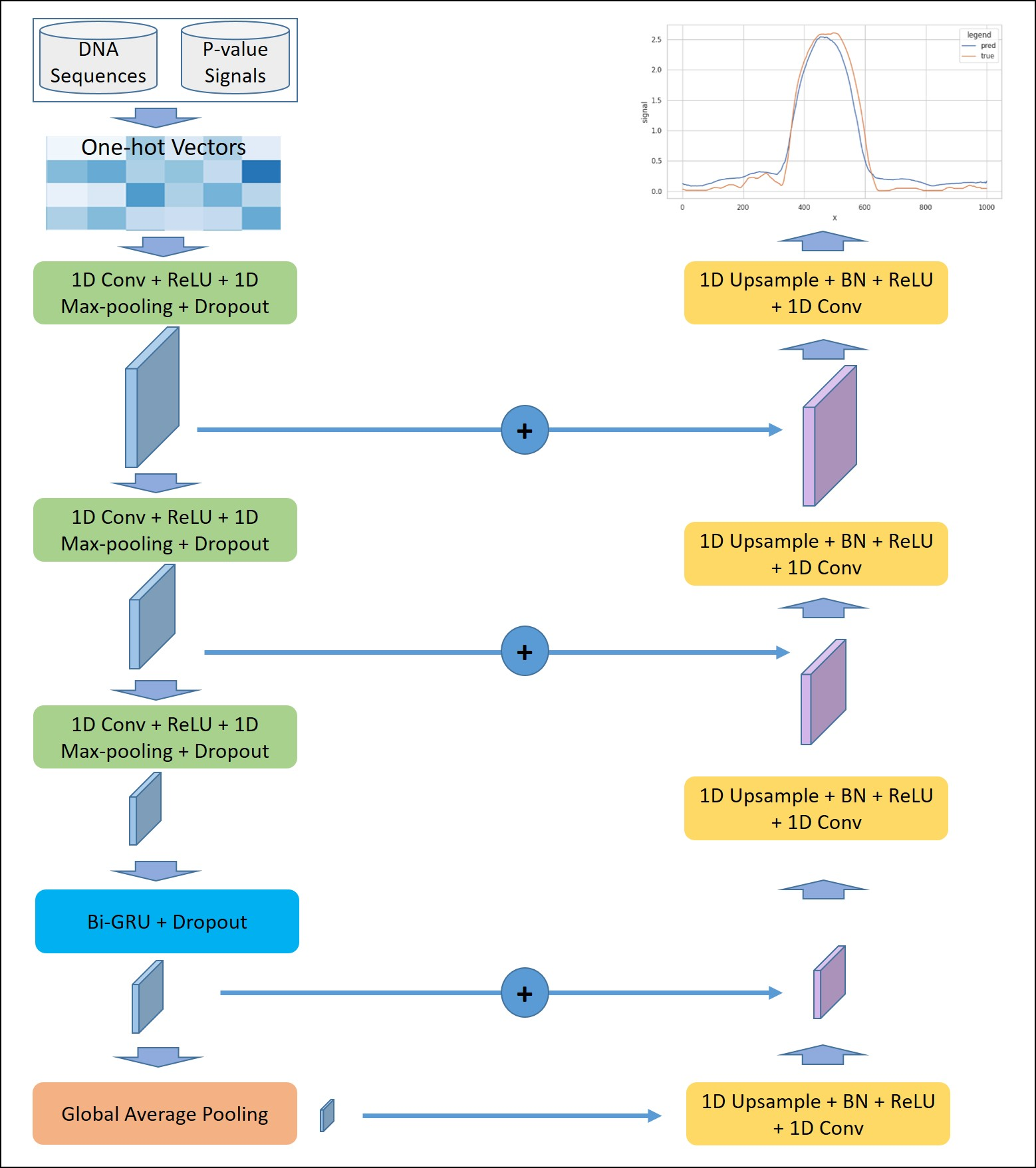

Supplement: S1 Fig — It is composed of i) an encoder architecture (left) for down-sampling the inputs and extracting sequence-specific features, ii) a decoder architecture (right) for up-sampling features and modeling the signal of each base, and iii) a skip architecture (middle) for transferring the position information in the encoder to the high-level features in the decoder. (TIF) [file pcbi.1009941.s002.tif]

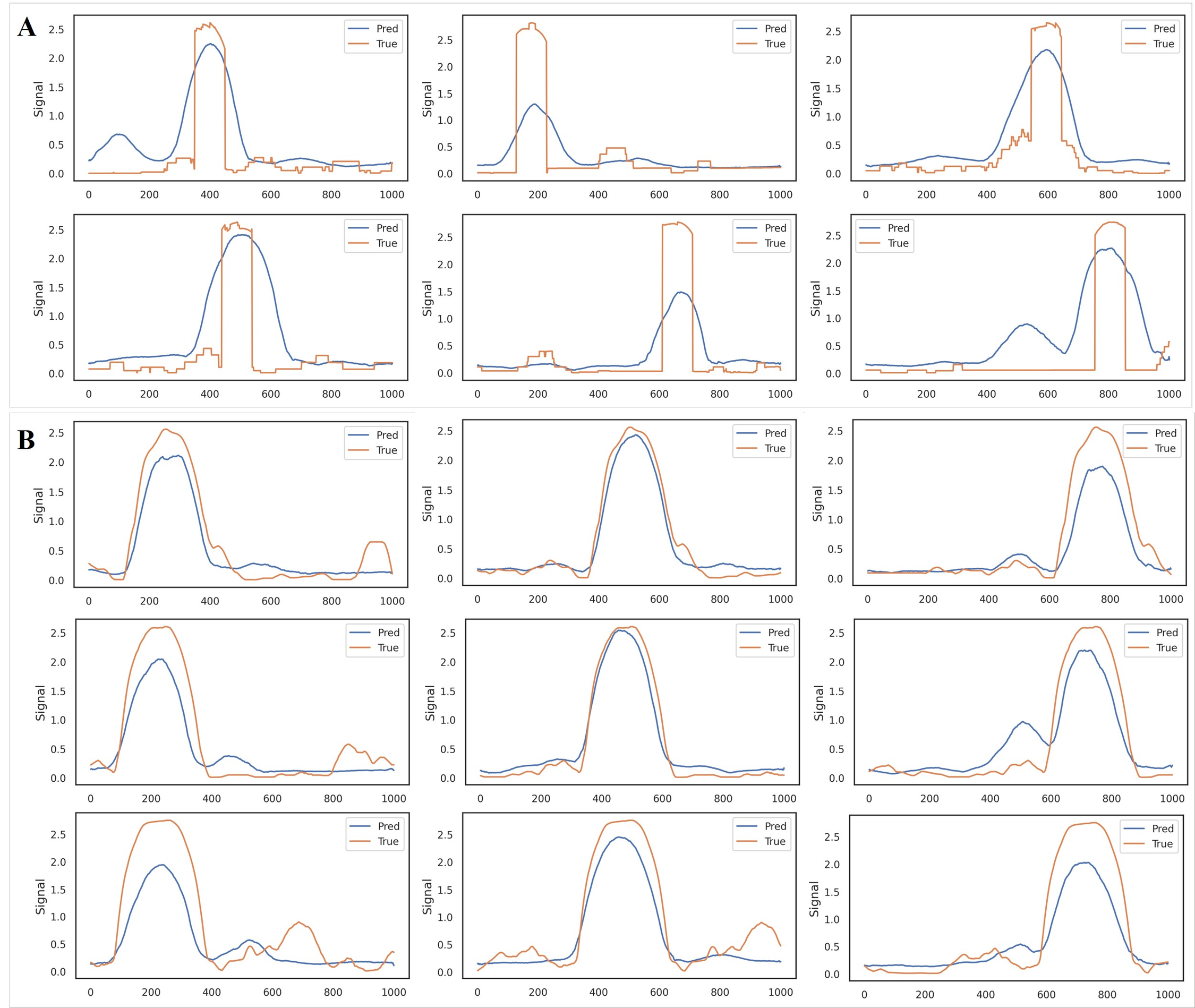

Supplement: S2 Fig — (A) The results of FCNsignal in predicting the base-resolution signals on the randomly inserted binding regions. (B) The results of FCNsignal in predicting the base-resolution signals on the shifted binding regions. (TIF) [file pcbi.1009941.s003.tif]

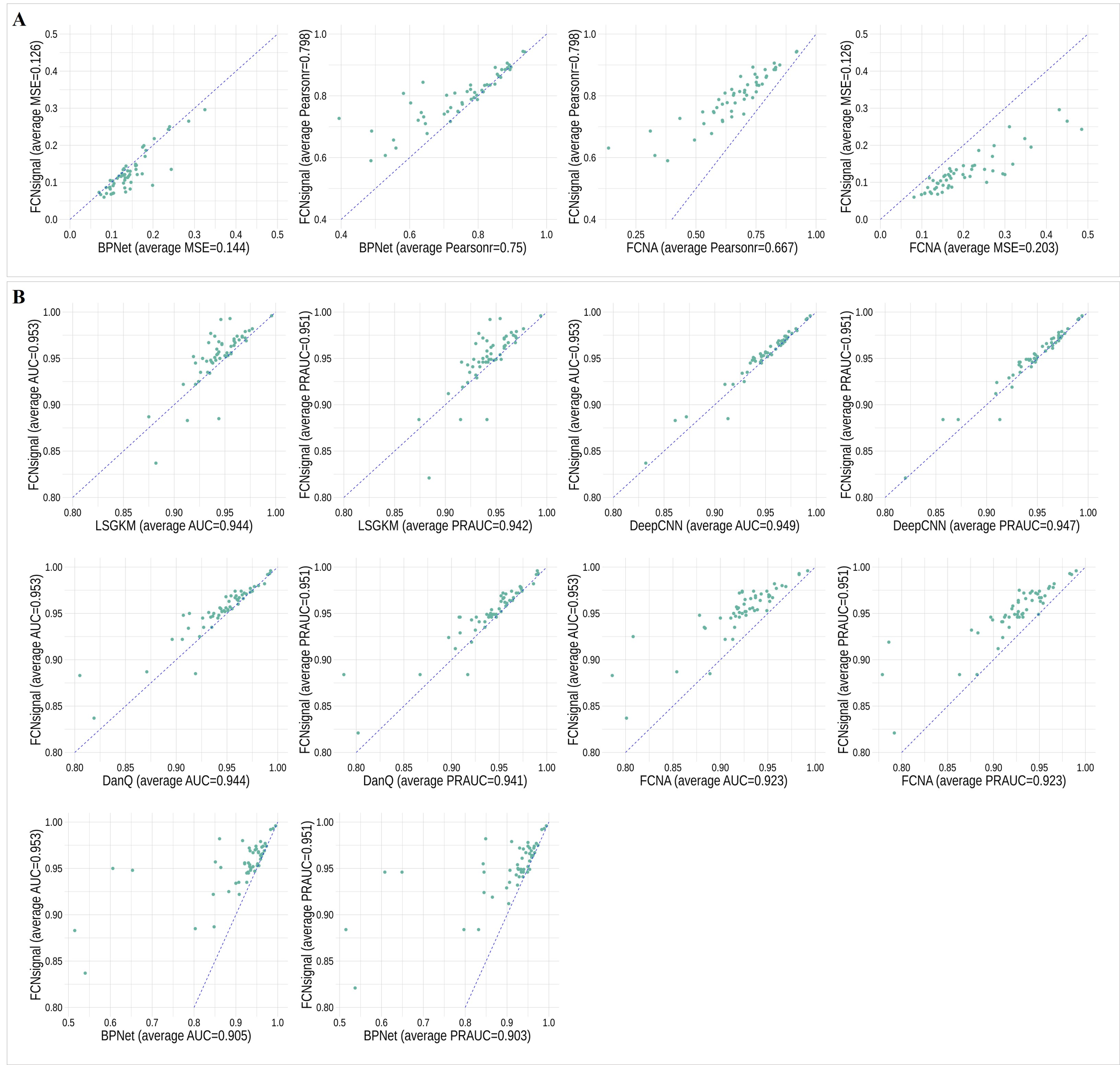

Supplement: S3 Fig — (A) Scatter plots for comparing FCNsignal with the two competing methods under the MSE and Pearsonr metrics. (B) Scatter plots for comparing FCNsignal with the five competing methods under the AUC and PRAUC metrics. (TIF) [file pcbi.1009941.s004.tif]

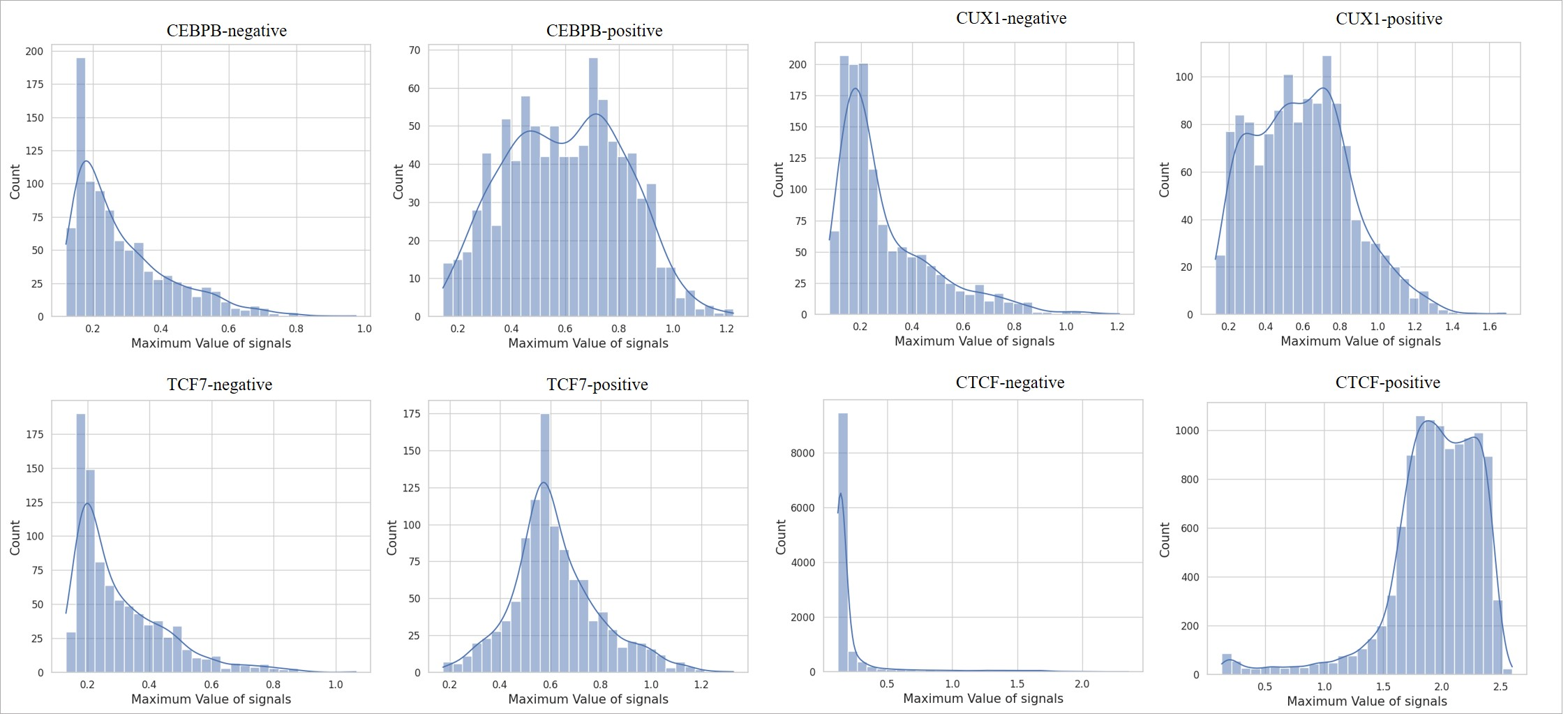

Supplement: S4 Fig — As we can see, the maximum values of signals in the positive and negative samples for CEBPB, CUX1, and TCF are difficult to distinguish while the ones for CTCF are easy to distinguish. (TIF) [file pcbi.1009941.s005.tif]

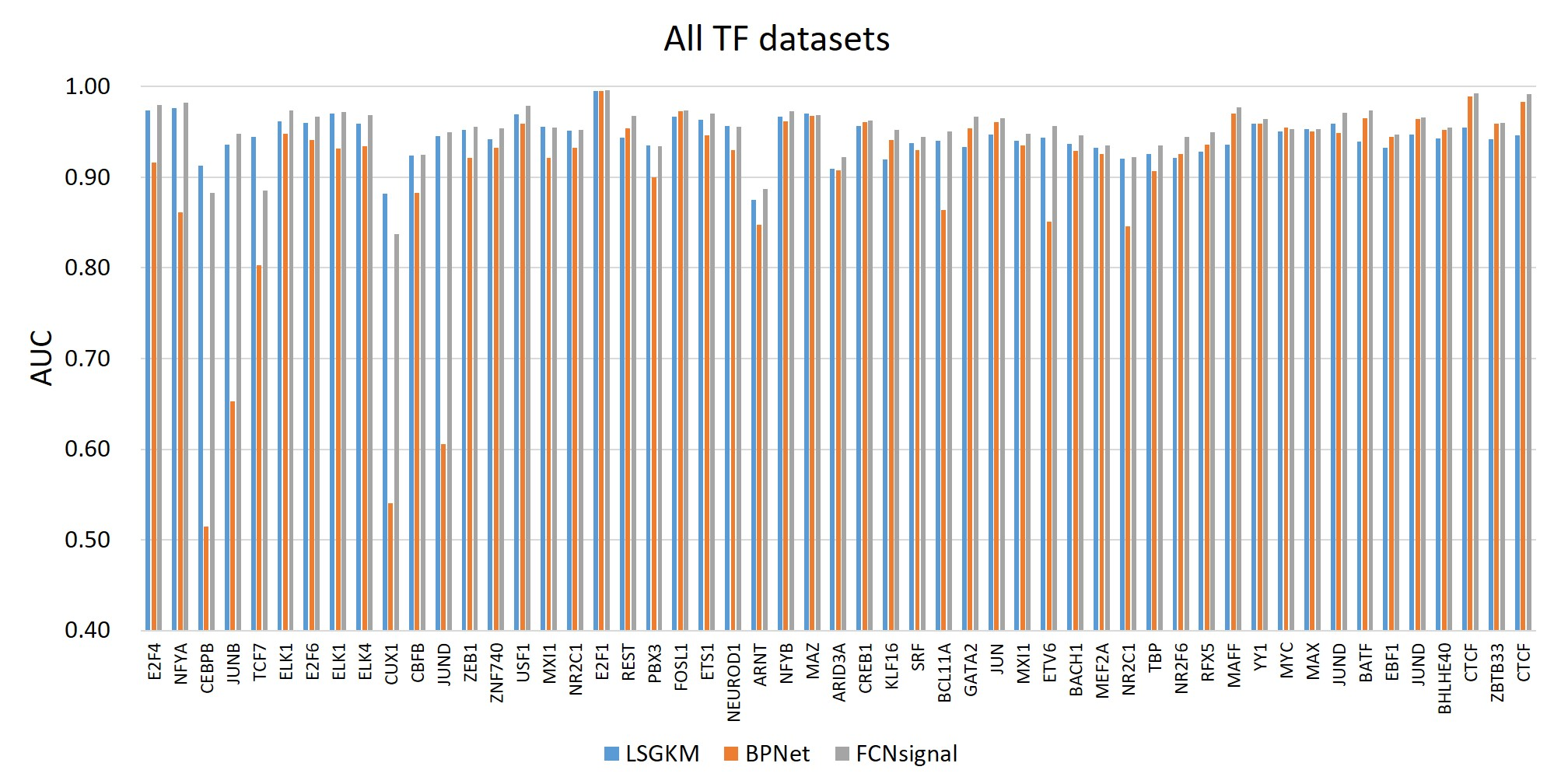

Supplement: S5 Fig — (TIF) [file pcbi.1009941.s006.tif]

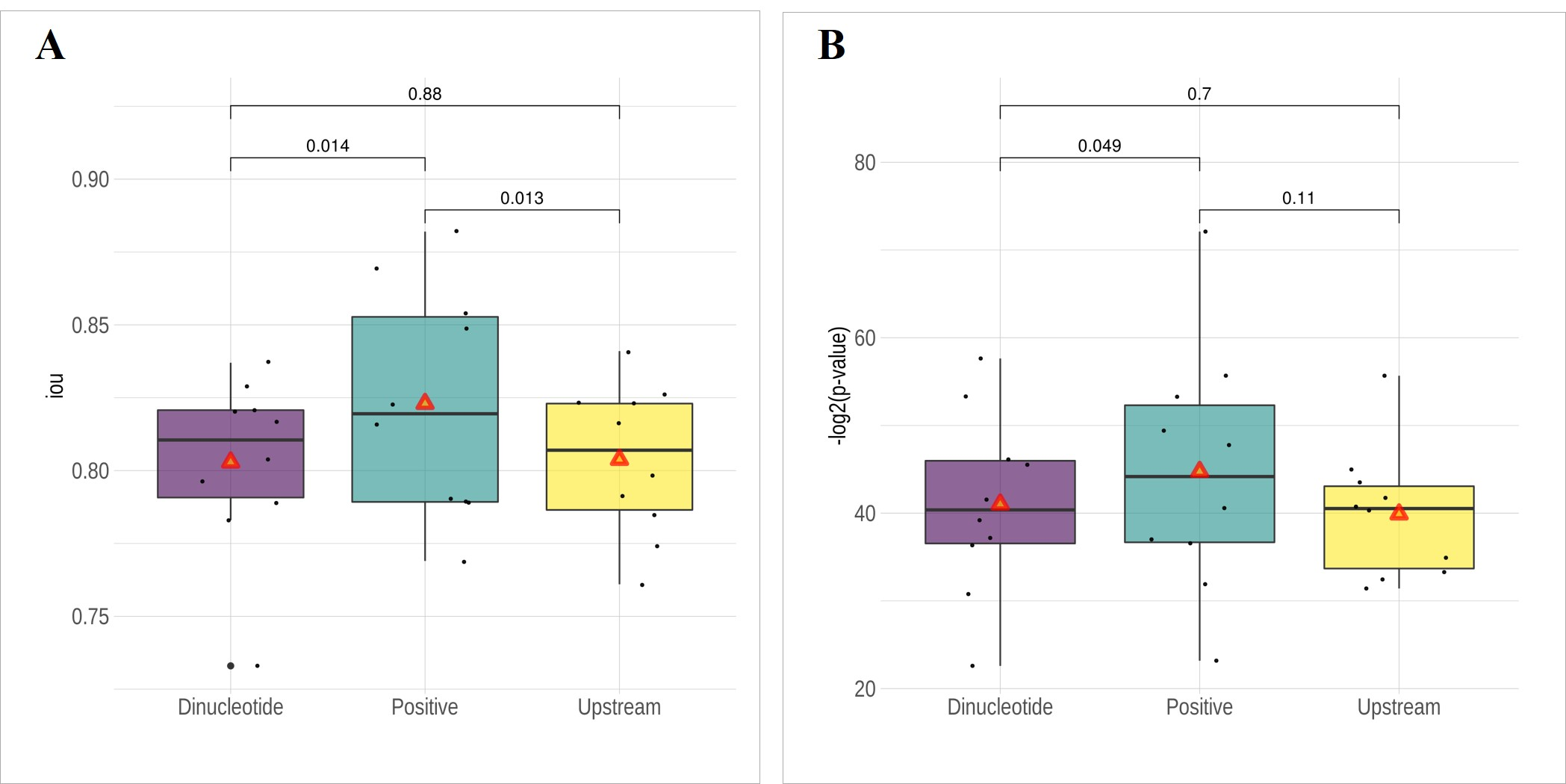

Supplement: S6 Fig — (A)The IOU (Intersection over Union) metric was used to test the performance of FCNA* in segmenting binding or non-binding sites. (B) The–log2(p-value) metric was used to test the performance of FCNA* in predicting binding motifs. The results show that FCNA* performs well on the negative sequences, inferring that FCNA* is extremely dependent on the PCMs so that it cannot distinguish positive sequences from negative sequences. The red triangles represent the average values. (TIF) [file pcbi.1009941.s007.tif]

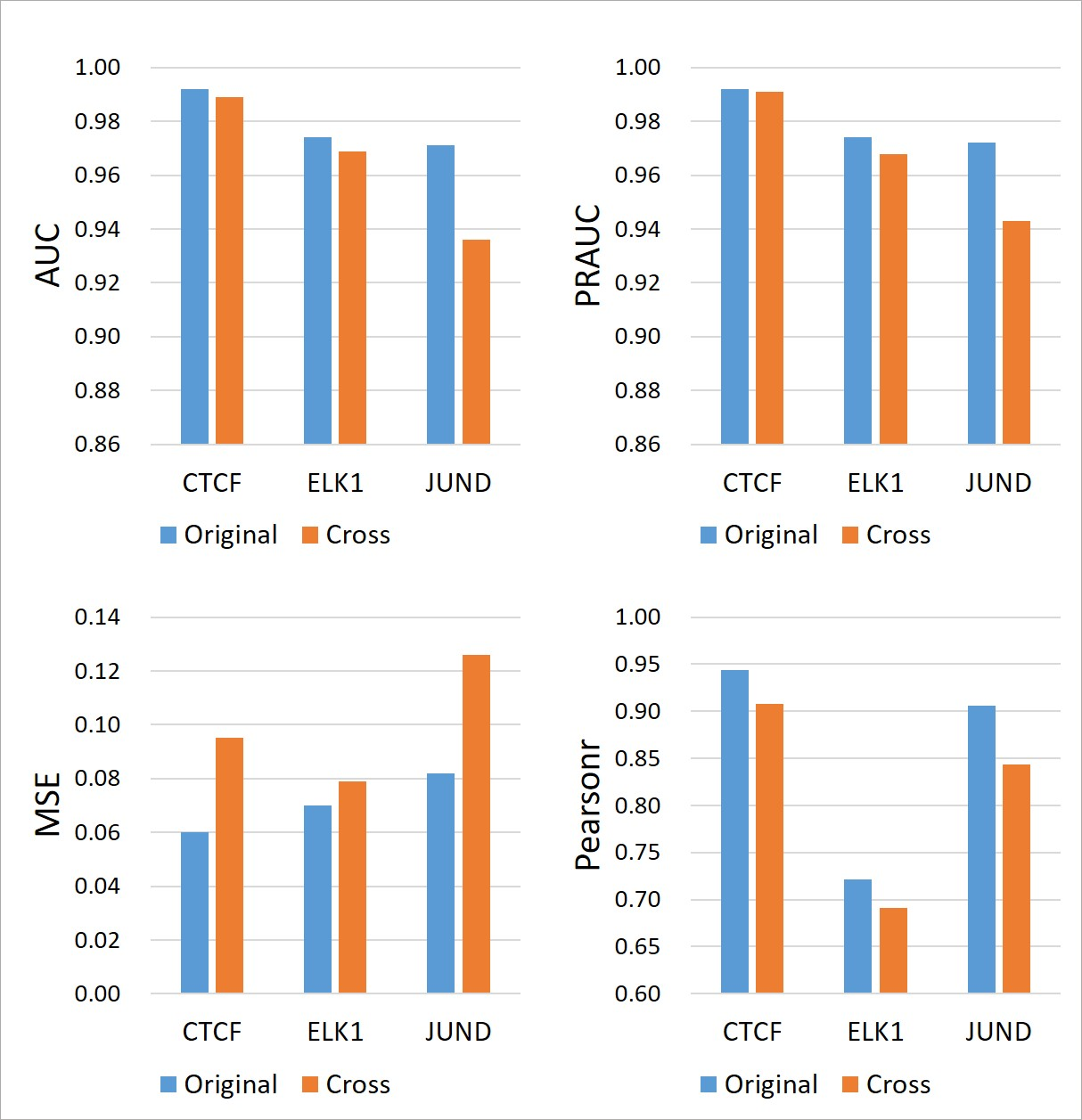

Supplement: S7 Fig — ‘Original’ means prediction on the same cell lines and ‘Cross’ means prediction on the different cell lines. (TIF) [file pcbi.1009941.s008.tif]

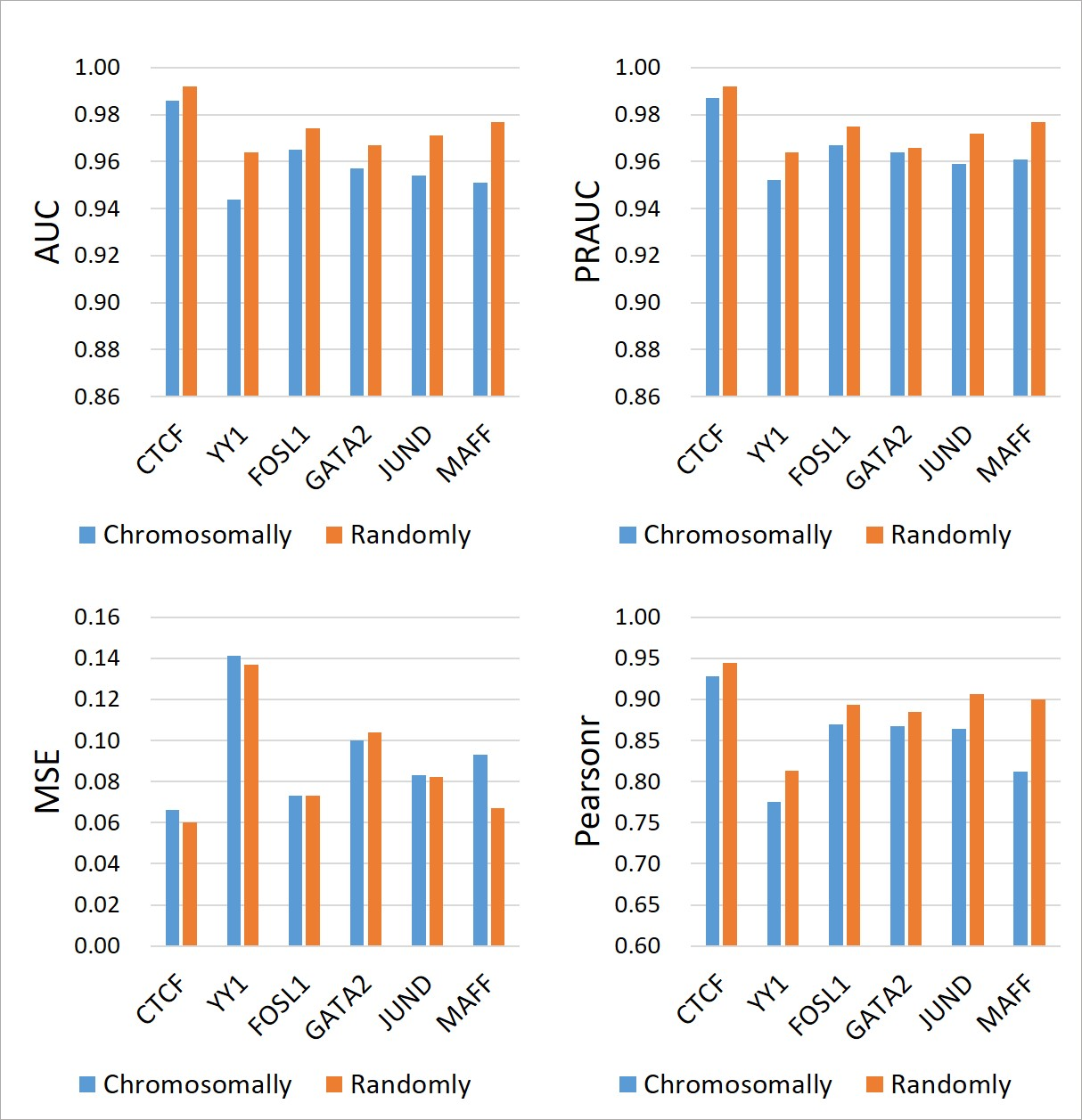

Supplement: S8 Fig — ‘Randomly’ means using the randomly-split strategy and ‘Chromosomally’ means using the chromosomally-split strategy. (TIF) [file pcbi.1009941.s009.tif]

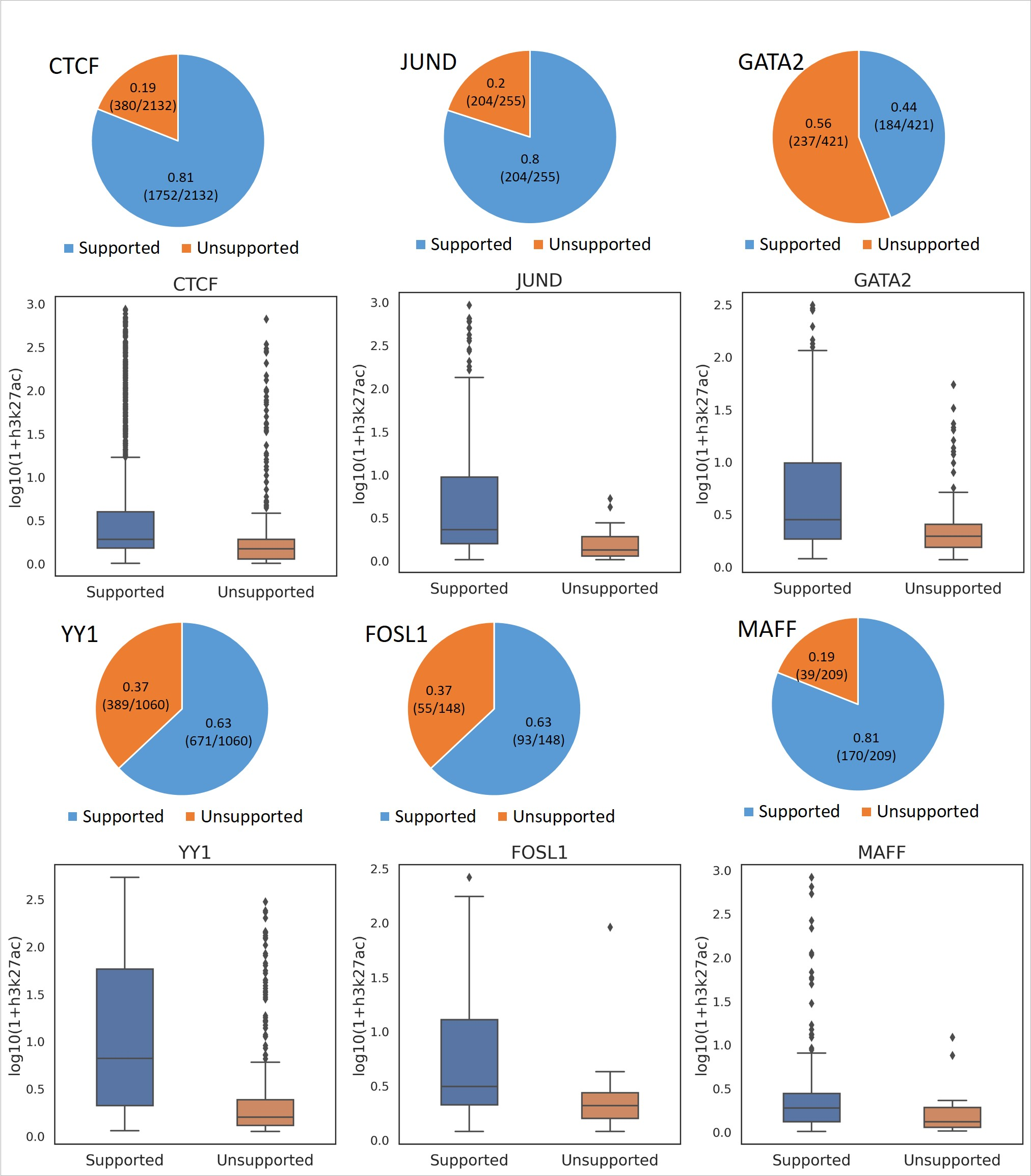

Supplement: S9 Fig — Most of these found regions are supported by the true peaks and the distribution of h3k27ac signals of the supported regions are significantly higher than that of the unsupported regions. (TIF) [file pcbi.1009941.s010.tif]

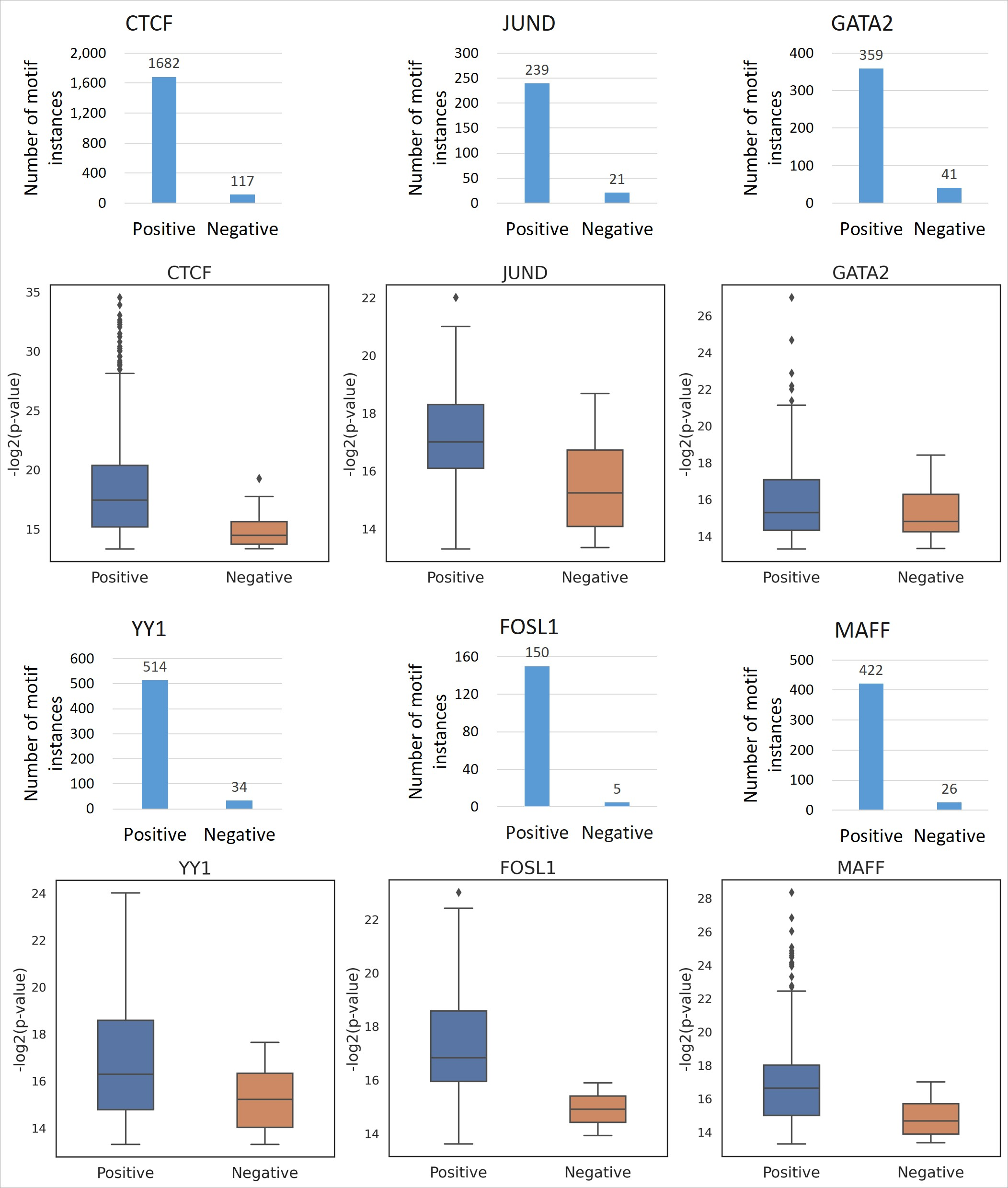

Supplement: S10 Fig — The number of motif instances discovered by FIMO on the located regions is much more than the ones found on the negative regions. Moreover, the–log2(p-value) values of the located regions are much higher than that of the negative regions. (TIF) [file pcbi.1009941.s011.tif]

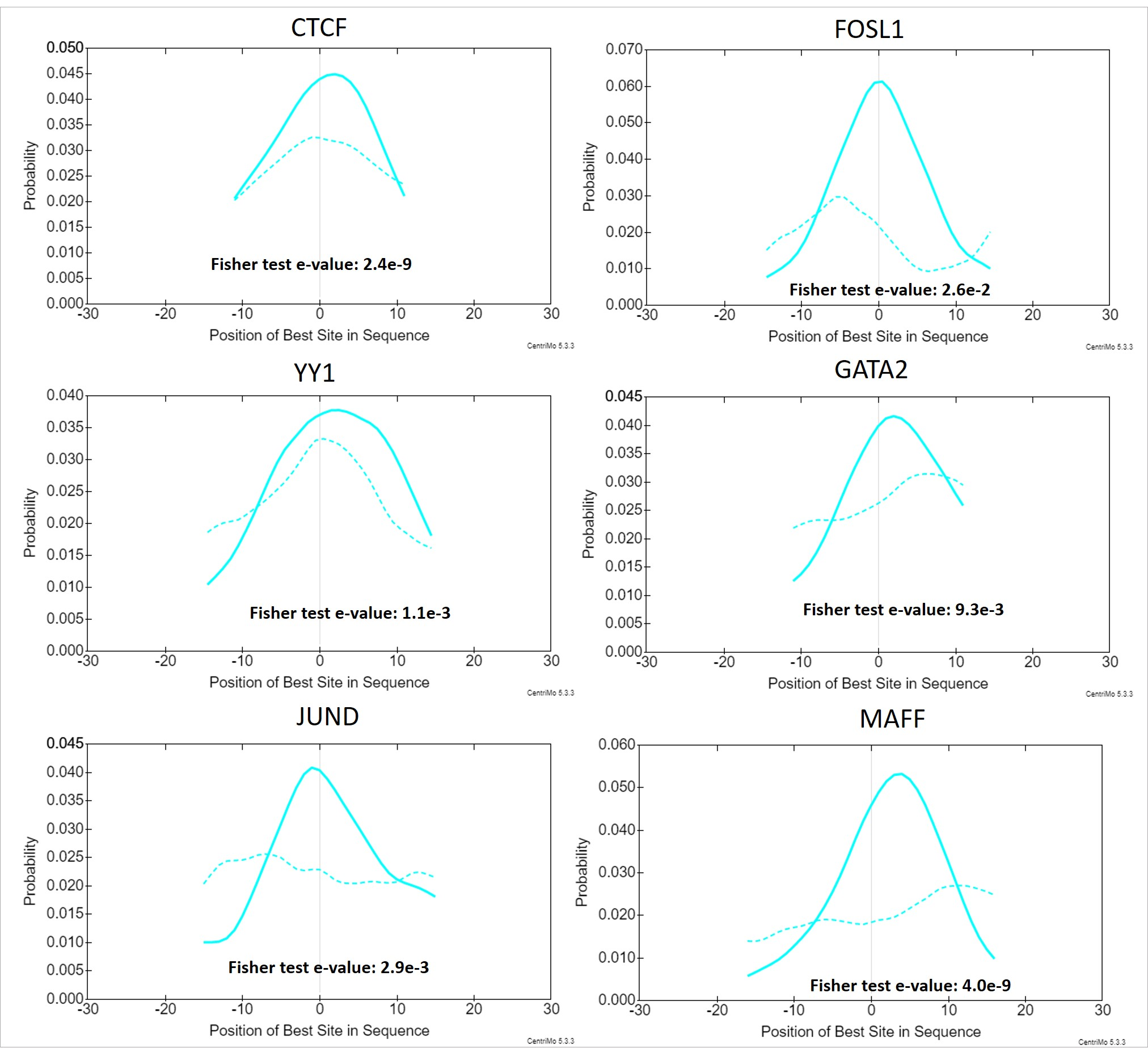

Supplement: S11 Fig — Motifs are more enriched on the located regions than that on the negative regions. The dash lines represent the results on the negative regions. (TIF) [file pcbi.1009941.s012.tif]

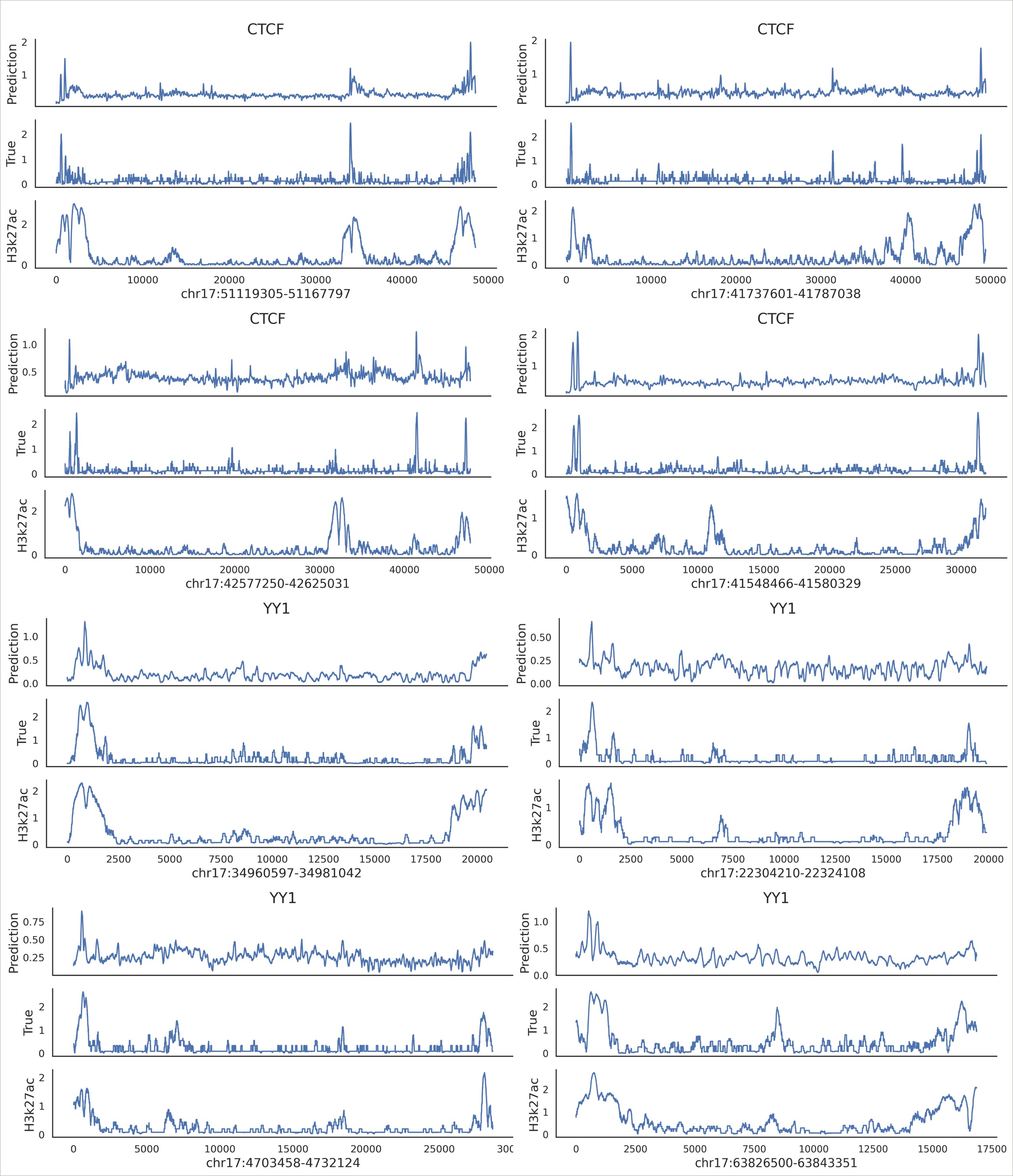

Supplement: S12 Fig — (TIF) [file pcbi.1009941.s013.tif]

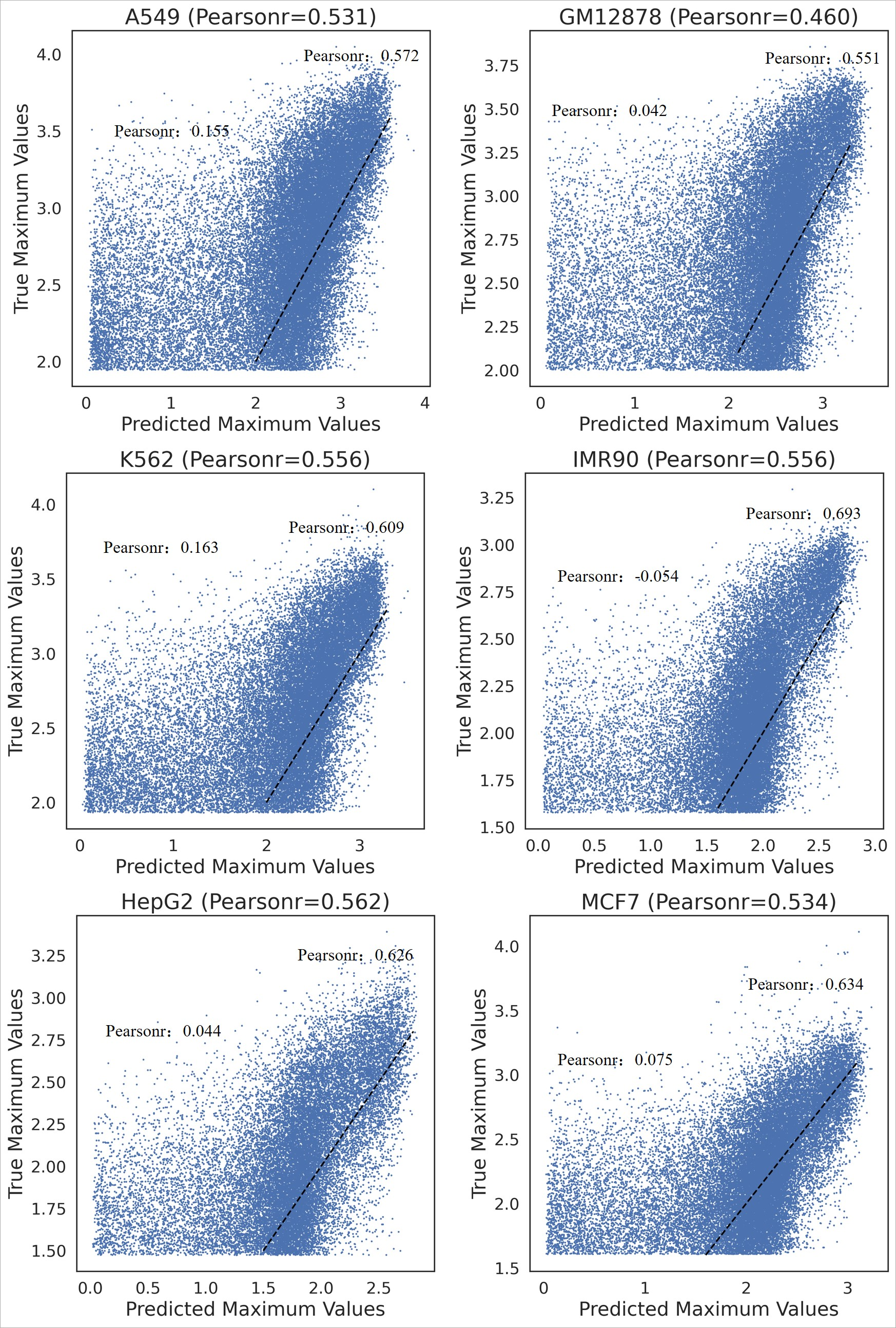

Supplement: S13 Fig — As we can see, FCNsignal performs well for DNA sequences with high openness values (mean Pearsonr: 0.62) but poorly for ones with low openness values (mean Pearsonr: 0.07). (TIF) [file pcbi.1009941.s014.tif]

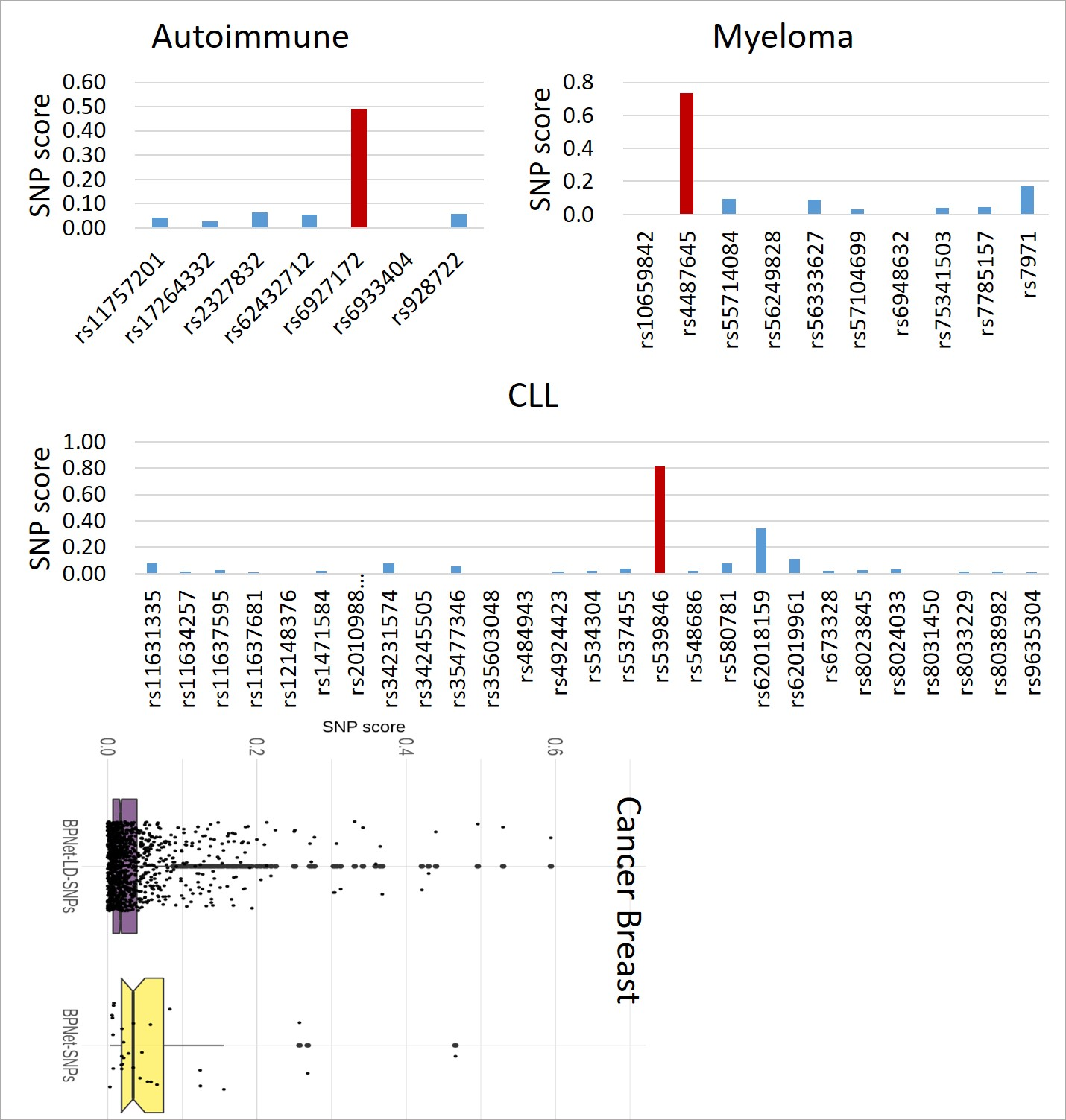

Supplement: S14 Fig — The risk variants for the myeloma, pan-autoimmune, and CLL are rs4487645, rs6927172, and rs539846 respectively. (TIF) [file pcbi.1009941.s015.tif]

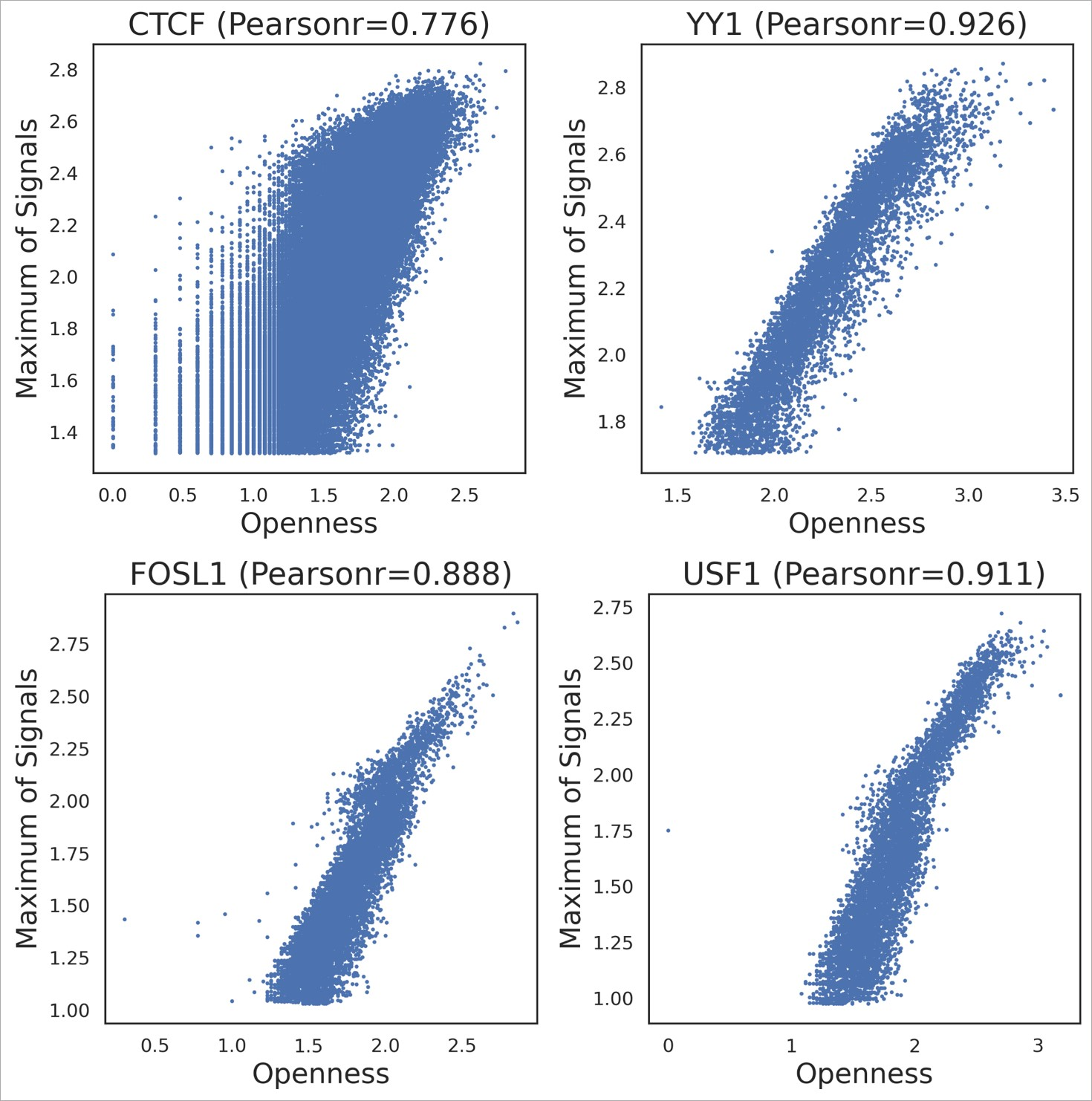

Supplement: S15 Fig — The results are 0.776, 0.926, 0.888, and 0.911 respectively, suggesting that the maximum values of signals can reflect the openness degree of TF-DNA binding. (TIF) [file pcbi.1009941.s016.tif]
